# Pathogenicity and functional impact of non-frameshifting insertion/deletion variation in the human genome

**DOI:** 10.1371/journal.pcbi.1007112

**Published:** 2019-06-14

**Authors:** Kymberleigh A. Pagel, Danny Antaki, AoJie Lian, Matthew Mort, David N. Cooper, Jonathan Sebat, Lilia M. Iakoucheva, Sean D. Mooney, Predrag Radivojac

**Affiliations:** 1 School of Informatics, Computing, and Engineering, Indiana University, Bloomington, Indiana, United States of America; 2 Department of Psychiatry, University of California San Diego, La Jolla, California, United States of America; 3 Center for Medical Genetics, School of Life Sciences, Central South University, Changsha, China; 4 Institute of Medical Genetics, Cardiff University, Cardiff, United Kingdom; 5 Department of Biomedical Informatics and Medical Education, University of Washington, Seattle, Washington, United States of America; 6 Khoury College of Computer Sciences, Northeastern University, Boston, Massachusetts, United States of America; University of Maryland Baltimore County, UNITED STATES

## Abstract

Differentiation between phenotypically neutral and disease-causing genetic variation remains an open and relevant problem. Among different types of variation, non-frameshifting insertions and deletions (indels) represent an understudied group with widespread phenotypic consequences. To address this challenge, we present a machine learning method, MutPred-Indel, that predicts pathogenicity and identifies types of functional residues impacted by non-frameshifting insertion/deletion variation. The model shows good predictive performance as well as the ability to identify impacted structural and functional residues including secondary structure, intrinsic disorder, metal and macromolecular binding, post-translational modifications, allosteric sites, and catalytic residues. We identify structural and functional mechanisms impacted preferentially by germline variation from the Human Gene Mutation Database, recurrent somatic variation from COSMIC in the context of different cancers, as well as de novo variants from families with autism spectrum disorder. Further, the distributions of pathogenicity prediction scores generated by MutPred-Indel are shown to differentiate highly recurrent from non-recurrent somatic variation. Collectively, we present a framework to facilitate the interrogation of both pathogenicity and the functional effects of non-frameshifting insertion/deletion variants. The MutPred-Indel webserver is available at http://mutpred.mutdb.org/.

## Author summary

An individual genome contains around ten thousand missense variants, hundreds of insertion/deletion variants, and dozens of protein truncating variants. Among them, non-frameshifting insertion and deletion variants exhibit diverse impact on protein sequence, encompassing alterations from a single residue to the deletion of entire functional domains. Although the majority of revealed insertion/deletions have unknown phenotypic consequences, computational variant effect prediction methods are less well-described for such variation. To this end, we develop MutPred-Indel, a machine learning method to predict the pathogenicity of non-frameshifting insertion/deletion variation and, in addition, highlight structural and functional mechanisms potentially impacted by a given variant. We identify several functionally important molecular mechanisms that are impacted differently among germline, de novo, and somatic variation in contrast to putatively neutral variation. MutPred-Indel is shown to have strong performance in pathogenicity prediction and potential to identify impacted molecular features, which collectively facilitates a deeper understanding of non-frameshifting insertion/deletion variation.

This is a *PLOS Computational Biology* Methods paper.

## Introduction

Insertion and deletion events comprise a diverse category of genetic variation that result in a range of phenotypic and molecular effects [1, 2]. In an individual genome, the dozens of sequence-retaining insertion, deletion and complex indel variants, referred to here collectively as non-frameshifting insertion/deletion variants or simply “indels”, are significantly less well-studied than single nucleotide substitutions. Non-frameshifting insertion/deletion variants result in the gain or loss of a number of nucleotides divisible by three, such that the reading frame of the mRNA is not disrupted. The resultant mutant protein sequence differs from the wildtype with the addition and/or deletion of one or more amino acid residues. In this work, three types of protein-coding insertion/deletion variants are discussed: insertions, deletions, and complex indel variants. The less abundant complex indel variants arise from events where both deletion and insertion events occur in tandem, and in this work comprise both deletion-insertion and complex substitution variants.

The phenotypic effects of a non-frameshifting insertion/deletion variant arise as a consequence of disrupted protein function and impact upon biological pathways. Variants affecting residues that participate in essential molecular events such as in protein-protein interaction interfaces or catalytic sites are more likely to be pathogenic. However, beyond pathogenicity, characterization of phenotypically impactful variant can extend to the molecular mechanisms by which protein function is altered. Computational methods to characterize the functional impact of missense variants have been diverse, including protein binding, post-translational modification, and stability [3–11]. In addition, several databases are available to support the analysis of missense variants [12–17]. By contrast, there are limited studies that utilize computational methods to assess the impact of insertion/deletion variation on protein function. Previously, phenotypically neutral sequence-retaining insertion/deletion variants have been found to segregate in disordered regions [18, 19] and small indels in the coil region have been shown to result in differences in binding affinity and gene expression [20]. Lin et al. evaluated the functional impact of insertion/deletion variation observed in the 1000 Genomes Project populations [21], finding enrichment in N- and C-terminal regions, coil, and disorder, as well as depletion in helix and strand secondary structure. In addition to germline variants, somatic microsatellite indel hotspots have been used to discover putative cancer driver genes [22]. Further analyses of somatic variation have identified complex indel variants in cancer genes that were almost entirely overlooked in previous analyses [23]. Collectively, these findings illustrate the diverse sources and implications of sequence-retaining insertion/deletion variation, particularly in cancer.

Computational methods to predict the consequences of genetic variation are well-suited to analyze the deluge of genetic information yielded by modern sequencing technologies [24, 25]. Such predictors generally focus on the pathogenicity of individual variants, rather than molecular impact or fine-grained phenotypic consequences. Computational methods to assess somatic variation largely seek to identify driver mutations, a small subset of variants that initiate or promote cancer growth. Methods to identify missense cancer driver mutations are diverse methodologically, utilizing known and predicted structural features including solvent accessibility, backbone flexibility, as well as helix, strand, and loop secondary structure [26–29]. Although methods show promise in the identification of cancer driver mutations, there are limited large-scale functional analyses of somatic variants. Previous work has found enrichment for amino acid substitutions that impact phosphorylation and other post-translational modification sites [30–32] as well as protein interfaces [33, 34] in somatic mutations compared to neutral controls.

In addition to curated locus-specific databases, such as ClinVar [35], computational methods serve to assess the pathogenicity of uncharacterized insertion/deletion variants [35, 36]. Previously developed methods trained specifically to predict the pathogenicity of non-frameshifting insertion/deletion variants are summarized in Table 1 [18, 37–40]. In addition, two alternative computational methods, CADD and PROVEAN [41, 42] generate pathogenicity prediction scores via a general prediction method, in addition to other types protein-coding variation. Many methods rely on the positive-unlabeled learning framework, wherein the negative class of neutral variants are a curated subset of putatively neutral variants from large-scale sequencing projects. To cleanse potentially pathogenic variation from sequencing project data many computational methods omit variants with low allele frequency and/or insertion/deletion size. The shown methods predominantly utilize pathogenic variation derived from a manually curated database of pathogenic variants, the Human Gene Mutation Database (HGMD) [36].

**Table 1:** Methods to assess the impact of non-frameshifting insertion/deletion variants.

		Training Data		Performance		
	Model	Pathogenic	Neutral	Balanced accuracy	Accuracy	AUC
DDIG-in [18]	SVM	HGMD	1000 GP	NA	0.83	0.89
KD4i [38]	Inductive Logic Programming	UniProtKB [43]		NA	0.78	NA
Zhang et al. [39]	Random Forest	HGMD	1000 GP	NA	0.88	NA
VEST-Indel [40]	Random Forest	HGMD	ESP6500	0.82-0.90	NA	NA
MutPred-Indel	Neural network	HGMD	gnomAD	0.81	0.83	0.91

In this study, we use predictive methods to assess the functional mechanisms impacted by non-frameshifting insertion/deletion variation and highlight mechanisms that are recurrently impacted by pathogenic and putatively neutral insertion/deletion variants. Next, we derive a method to identify structural and functional mechanisms that are significantly impacted by an individual non-frameshifting insertion/deletion variant compared to a background of putatively neutral variation. We construct a machine-learning method to predict the pathogenicity and functional impact of non-frameshifting insertion/deletion variation utilizing sequence-level, evolutionary, and predicted functional features on training data designed to mitigate the pervasive biases of stringent variant filtering. We show that the method exhibits robust predictive performance both in cross-validation and on an independent test set of cancer driver mutations. Finally, we highlight the structural and functional mechanisms impacted by somatic, disease-causing germline, and putatively neutral insertion/deletion variants.

## Materials and methods

### Training data sets

Disease causing sequence-retaining insertion, deletion, and complex indel variants were obtained from the Human Gene Mutation Database (HGMD), professional version 2017.1 [36]. For brevity, we will refer to the set of non-frameshifting insertion, deletion, and complex indel variants collectively as “insertion/deletion variants” or simply “variants” for the remainder of the text. Putatively neutral insertion/deletion variants were derived from the Genome Aggregation Database (gnomAD) [44]. In the process of collecting data, variants from gnomAD with Allele Count (AC) annotation of zero were considered to be of low quality and removed from the training data. Variants annotated within gnomAD with AC equal to one were similarly removed to reduce noise that may arise as a consequence of variants called in error. For each variant, the wild-type and mutant protein sequence were determined using ANNOVAR [45]. The number of variants considered in model training are described in Table 2. In total, the training data comprised 5606 single residue deletions, 1033 single residue insertions, 2427 multi-residue insertions, 3052 multi-residue deletions, and 1253 complex indel variants.

**Table 2:** Number of variants (proteins) in the training data set.

	Disease	Neutral	Total
Insertion	653 (370)	1774 (946)	2427 (1307)
Deletion	3012 (1052)	5646 (2162)	8658 (3143)
Complex indel	1014 (528)	239 (209)	1253 (733)
Total	4679 (1296)	7659 (2392)	12338 (3597)

### Somatic test sets

To assess the utility of MutPred-Indel on an alternative source of pathogenic variation, we apply the tool to two sets of putatively damaging somatic variants. First, we extract insertion/deletion variants from the Catalogue Of Somatic Mutations In Cancer (COSMIC) genome-wide screen data set (v85) [46]. For analysis of the structural and functional impact of somatic variants, the COSMIC primary histology annotations are used to retain histology types with at least 500 variants, and exclude variants with “Other” or “Not specified” annotation. In this work, we define recurrent somatic mutations as those which impact the same residue more than once by either missense or non-frameshifting insertion/deletion variants in the COSMIC dataset, a modification of the methods described in [47, 48]. Next, manually curated driver insertion/deletion variants are derived from the DataBase of Cancer Driver InDels (dbCID) [49]. The most confident variants from dbCID are retained for further analyses, those supported by in vivo experimental evidence. To ascertain excess of high scoring somatic variants in known cancer genes, we select Tier 1 cancer genes described in the Cancer Gene Census to represent the genes with high-quality documented relevance to cancer (*n* = 576) [50].

### De novo test set

We assess the performance of MutPred-Indel on de novo non-frameshifting insertion/deletion variants curated from 2650 families (2703 cases, 2009 controls) affected by autism spectrum disorder (ASD) from the REACH Project [51] and the Simons Simplex Collection (SSC) [52]. De novo genetic variants, which occur in offspring but not in parents, arise from spontaneous mutations in either the parent’s germline or early in embryonic development. Detecting de novo variants is challenging, as a false positive call in an offspring can appear to be an apparent de novo variant. Without filtering, the false discovery rate for de novo variants can be as high as 80% [53]. A naive approach to filter putative de novo variants would rely on heuristic hard filters that negatively affects sensitivity. We and others [54] have relied on machine learning as a replacement for hard filters for de novo variant calling. Variant calls were produced using HaplotypeCaller with variant score recalibration using GATK v3.5. Variant calling for the REACH cohort were generated with respect to family as described previously [51], while families from the SSC were jointly called by batch. We then extract all de novo variants and generate exonic function annotations with ANNOVAR [45]. Variants were retained if the exonic annotation was either NFS insertion, deletion, or block substitution. We remove variants if the derived allele was present at or above a 1% allele frequency in the gnomAD database [44]. Variants with the same genomic position and alternate allele were removed, as these are likely common variants that were mis-genotyped in the parents. After these filters, there are 1217 candidate de novo insertion/deletion variants in 827 offspring (506 cases, 321 controls).

Filtering of de novo indels from the VCF files generated by HaplotypeCaller was performed using a random forest classifier (pyDNM) that was trained on a combination of simulated and validated de novo indels. The false discovery rate of the final call set based on experimental validation is 3% (Lian, Sebat et al, in preparation). Applying the pyDNM classifier resulted in 183 de novo variants called as true positives in 169 offspring (98 cases, 71 controls). We generate pathogenicity scores for the 168 variants for which ANNOVAR was able to obtain the wildtype and mutant protein sequences.

### Feature engineering

Features to describe each variant incorporated properties of the wildtype protein sequence, consisting of evolutionary conservation, predicted structural and functional features, and general sequence features. The general sequence features included the relative position of the variant in the protein sequence, the number of residues inserted by the variant, and number of residues deleted by the variant. Next, we identified simple repeats and low-complexity regions by encoding (1) the frequency of each amino acid in a ten-residue window on either side of the variant and (2) the length of single amino acid repeat at the variant site, where length equals 1 if the variant does not lie in a repeat.

The evolutionary features included the position-specific scoring matrix (PSSM), sequence conservation indexes, and the number of homologs in the human and mouse genomes. The PSSMs were generated by running PSI-BLAST with default parameters against the nr database [55]. To derive conservation indexes, we applied AL2CO [56] on the UCSC Genome Browser 46-species alignment [57]. We calculated both normalized and unnormalized versions of the nine available conservation indexes derived from AL2CO over three alignments: the full 46-species alignment, the mammalian alignment, and the primate-only alignment. For deletions and complex indel variants, conservation was encoded as the maximum of these AL2CO-derived conservation indexes over the range of amino acids deleted by the variant. For insertions, the maximum of the AL2CO-derived indexes was taken over a window of residues starting from the first residue prior to the insertion site with window size set to be the number of inserted residues. For each protein sequence, we calculated the homolog-based features as the number of homologs in the human genome and homologs in the mouse genome at levels of sequence identity from 50 to 100 percent in intervals of 5 percent sequence identity, for a total of 10 counts per organism.

Computationally predicted structural and functional features included gene-level functional annotation and residue-level molecular and structural function. The gene-level features are predicted scores for the total of 2,132 Gene Ontology (GO) terms generated by the FANN-GO method [58]. At the residue level, we characterized the impact of the variant on predicted structural and functional properties utilizing the nearby region in the wildtype sequence. Predictive scores were used to count the residues within a window of the variant site that exhibit high structural and/or functional prediction scores. In this work, the features were encoded for window sizes of four and twenty residues to identify functional sites both in the immediate vicinity of the variant site and the broader surrounding regions of the protein sequence. Next, we encoded the number of residues in the entirety of the protein sequence that are predicted to exhibit each of the structural and functional features in Table 3. For each feature, we ascertained predicted functional residues utilizing a confident score threshold defined for each predictive model (corresponding to 10% false positive rate).

**Table 3:** Predicted structural and functional features. * indicates in-house predictors.

Property category	Predicted features
Structure and dynamics	Helix*, strand*, loop*, Intrinsic disorder [59], B-factor [60], Relative solvent accessibility*, Coiled-coil region*
Signal peptide and transmembrane*	N- and C-termini of signal peptide, signal helix, signal peptide cleavage site, transmembrane segment, cytoplasmic and non-cytoplasmic loops
Macromolecular binding	DNA*, RNA*, Protein-protein interaction (PPI)*, PPI hotspots*, Molecular Recognition Features (MoRFs)*, Calmodulin-binding [61]
Metal-binding*	Cd; Ca; Co; Cu; Fe; Mg; Mn; Ni; K; Na; Zn
Post-translational modification (PTM) [62]	Acetylation, ADP-ribosylation, Amidation, Carboxylation, Disulfide linkage, Farnesylation, Geranylgeranylation, Glycosylation (C, N and O-linked), GPI anchor amidation, Hydroxylation, Methylation, Myristoylation, N-terminal acetylation, Palmitoylation, Phosphorylation, Proteolytic cleavage, Pyrrolidone carboxylic acid, Sulfation, SUMOylation, Ubiquitylation
Other	Allosteric residues*, Catalytic residues*, Motifs [63, 64]

### Predictor development and evaluation

Each pathogenicity predictor was developed with the Matlab 2016b Neural Network Toolbox as an ensemble of one hundred bagged two-layer feed-forward neural networks, where the following training parameters were not varied between alternative models. Each network had ten hidden units and employed balanced training with uniform random sampling of the majority class. Minimal feature reduction was performed, consisting of a two-sample t-test with a minimally restrictive 0.5 P-value threshold and principal component analysis with 99% retained variance applied on z-score normalized data. Finally, model training utilized the resilient propagation method with 25% of training data set aside for the validation set [65].

Performance of the models developed here are shown as the area under the Receiver Operating Characteristic (ROC) curve (AUC) derived from scores generated in 10-fold cross-validation. To illustrate the influence of protein-based features, we compared model performance based upon per-protein and per-cluster cross-validation protocols in training. In per-protein cross-validation, all variants within the same protein were either included in the test or training set partition. Per-cluster cross-validation retained variants from proteins with at least 50% sequence identity in the same partition. The per-cluster cross-validation method estimated performance when MutPred-Indel is applied to proteins that are dissimilar to the training set. Next, we assessed the performance of MutPred-Indel without low frequency gnomAD variants removed from the training data, utilizing variant frequency annotations included in the gnomAD database. In particular, the allele count annotation describes the number of times a particular allele has been observed in the gnomAD cohort. The alternative training set included variants where the AC value is exactly 1, effectively allowing for cases where only a single individual is heterozygous for the variant in the gnomAD database (*n* = 9,876 variants). Finally, to compare the importance of different feature sets on the final performance of MutPred-Indel, we estimated the performance in per-protein cross-validation of alternative models with individual sets removed from the training data, where the training parameters are identical to those described above.

### Significance of functional impact

To identify variants with significant impact on any particular functional mechanism, we defined an empirical P-value similar to the methodology employed in the initial MutPred publication [6]. Under this framework, for each feature listed in Table 3 the null distribution is defined by the functional disruption scores for the neutral training set. The P-value for functional impact of any particular variant is defined as the fraction of neutral variants with scores that are at least as high as the given value.

The above method relies on assumptions that each functional mechanism is equally likely to occur and equally likely to be disrupted in the null distribution. The assumption of equal distribution impacts the validity of P-value ranking among different mechanisms, and so to mitigate the effect of this assumption we adjust the P-values as

$$\begin{array}{c} P^{\prime }=\left(1-\alpha \right)\cdot P, \end{array}$$ (1)

where *P* is the P-value as defined above, *α* represents the frequency of a particular functional mechanism, and *P*′ will be referred to as the prior-corrected P-value. The P-value correction is drawn from the definition of false discovery rate (FDR),

$$\begin{array}{c} \text{FDR}=\frac{(1-\alpha )\cdot \text{FPR}}{\alpha \cdot \text{TPR}+(1-\alpha )\cdot \text{FPR}}, \end{array}$$ (2)

where FPR is the false positive rate and TPR is true positive rate. We consider the P-value to approximate the false positive rate, without considering the denominator.

The functional impact score per mechanism for each insertion/deletion variant is defined as the number of residues impacted by the variant which are confidently predicted to exhibit the functional mechanism. Here, we defined impacted residues to include the three amino acids on either side of the variant site, in addition to any residues that have been deleted. For each model described in Table 3, the thresholds for confident predictions were determined separately and correspond to a low false positive rate (10%). The null distribution of scores was derived from the training set of gnomAD variants, which have undergone minimal filtering to remove low frequency variants. The values of *α* for each mechanism were estimated using the AlphaMax algorithm [66].

### Enrichment of structural and functional impact

We define enrichment as a modification of the trend value described in Li et al. [31]. The enrichment value, *E*, is defined as

$$\begin{array}{c} E=\frac{F_{pathogenic}-F_{neutral}}{F_{pathogenic}+F_{neutral}}, \end{array}$$ (3)

where *F*_*pathogenic*_ and *F*_*neutral*_ are the fraction of canonical sequence variants in which the modified residues are predicted to exhibit the mechanism of interest in HGMD and gnomAD, respectively. Positive trend values indicate an excess of functional impact in residues impacted by pathogenic variants as compared to putatively neutral variants, whereas negative values indicate an excess of functional effect in the set of neutral variants. Significance is assigned with Fisher’s exact test after Bonferroni correction.

In the comparison of variants from HGMD and gnomAD, we utilized the structural and functional mechanisms impacted directly by the variant. For deletions and complex indel variants, we considered affected residues to be those amino acids deleted by the variant. For insertion variants, we approximated the affected region of the protein to be the two residues on either side of the insertion site.

### Functional impact of germline and somatic variants

To interrogate the characteristics of disease-causing variants compared to somatic variation, we contrasted the proportion of variants that significantly impact structural and functional mechanisms. Specifically, for each mechanism in Table 3, we identified non-frameshifting insertion/deletion variants that significantly impact (*P* < 0.05) a single residue with function prediction score above the confident threshold (10% FPR). Variants impacting more than one residue were not included in the functional analyses to ensure that the increased functional impact among the minority of longer insertion/deletion variants did not distort the conclusions. For functional analyses described here, each variant were considered once per individual in the canonical isoform, to mitigate the functional bias of including similar protein isoforms.

### Output format

For every variant, MutPred-Indel returns a pathogenicity prediction score between zero and one, where variants with scores close to one are more likely to be pathogenic. Three score thresholds can be used to classify variants as pathogenic or neutral at different values of false positive rate (FPR): 0.546 (10% FPR), 0.672 (5% FPR, recommended), 0.85 (1% FPR). We utilize the 10% FPR score threshold on the pathogenicity prediction scores determined through cross-validation to determine the accuracy and balanced accuracy shown in Table 1. In addition, MutPred-Indel returns the top five structural and functional mechanisms that are impacted by the variant with significant prior-corrected P-values less than 0.05.

### Comparison to previously developed insertion/deletion prediction methods

We compared the performance of MutPred-Indel against three currently available methods to assess insertion/deletion variation: DDIG-in, VEST-Indel and CADD. As we were unable to access source code or web implementation for the methods described by Bermejo et al. [38] and Zhang et al. [39], these methods are excluded from further analyses. The PROVEAN method was similarly removed from consideration, as we were unable to generate predictions for the majority of the test set. To maximize the test set, and as a consequence of the paucity of publicly available curated insertion/deletion variation, the test set was extracted from the MutPred-Indel training data derived from HGMD and gnomAD. We removed test set variants that were included in the training data of DDIG-in and VEST-Indel, a procedure which could not be repeated for the CADD training data. The MutPred-Indel scores utilized for this comparison are generated in per-protein cross-validation, which ensures that neither the variant nor any other variants within that protein sequence are utilized in the model underlying a given pathogenicity prediction, a constraint that has not been placed upon the other methods in this comparison. From this pool of variation, we randomly selected a balanced test set of one thousand pathogenic variants and one thousand putatively neutral variants from gnomAD.

## Results

### Properties of insertion/deletion variants

**Fig 1:**
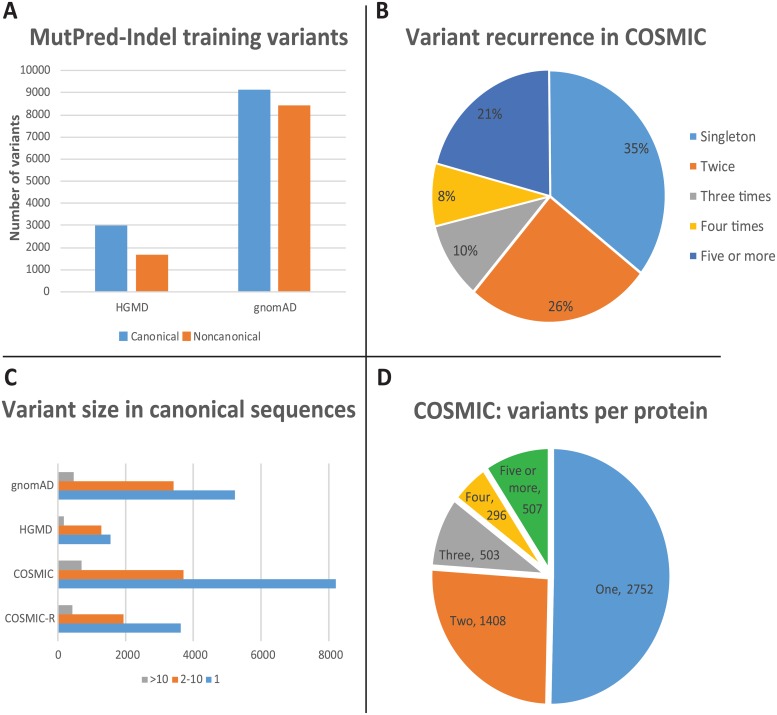
Characteristics of variants included in the functional analyses. (A) Training variants in canonical and noncanonical protein sequences. (B) Recurrently impacted residues in COSMIC. (C) Variant size in gnomAD, HGMD, COSMIC, and recurrent variants in COSMIC (COSMIC-R). Size of complex indels is the maximum of the number of amino acid residues inserted or deleted. (D) Variants per protein in COSMIC.

Factors that differentiate disease-causing and apparently neutral variation can shed light on the mechanisms underlying variant pathogenicity. In particular, we contrast the structural and functional mechanisms predicted to be impacted among variants in the training data. Fig 1A shows the number of training variants in canonical protein sequences retained for these analyses, representing 35-47% of the original training data.

**Fig 2:**
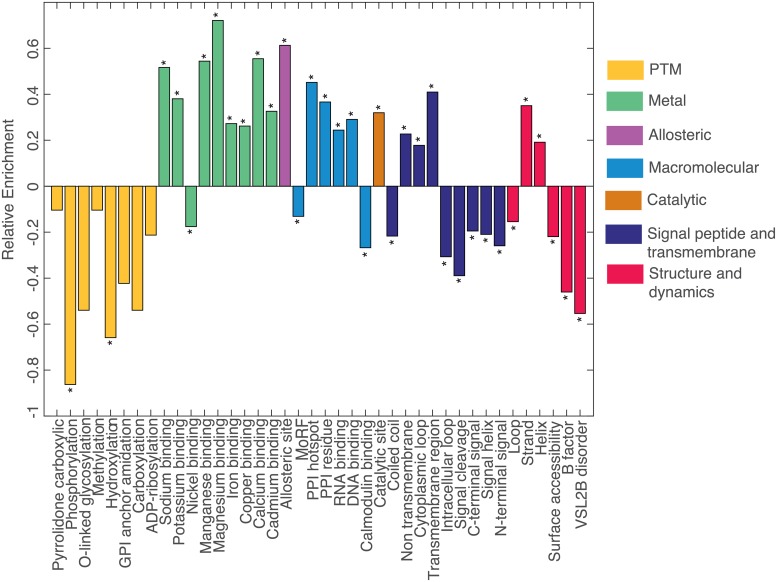
Relative enrichment of mechanisms impacted by pathogenic variants from HGMD compared to gnomAD. Negative trend values correspond to enrichment in putatively neutral variation. * indicates statistical significance after Bonferroni correction.

Mechanisms in Table 3 that exhibit relative enrichment in the training data are shown in Fig 2. Among these, we find enrichment for mechanisms associated with protein flexibility in neutral variants including disorder, MoRF, and B-factor, consistent with Khan et al. [19] and Zhao et al. [18], as well as with work characterizing missense variation [67–70]. The trend continues for surface accessible residues, which may be less likely to induce conformational changes than internal insertion/deletion variants. Pathogenic variation shows enrichment for impact upon critical protein functional residues such as catalytic and protein-protein interaction sites. Structural features exhibit further differentiation, we observe enrichment for loop regions in neutral variants whereas pathogenic variants show enrichment for helix and strand secondary structure. Collectively, these findings indicate that predicted structural and functional features have the potential to inform variant impact in addition to pathogenicity prediction.

#### Characteristics of somatic variants from COSMIC

**Fig 3:**
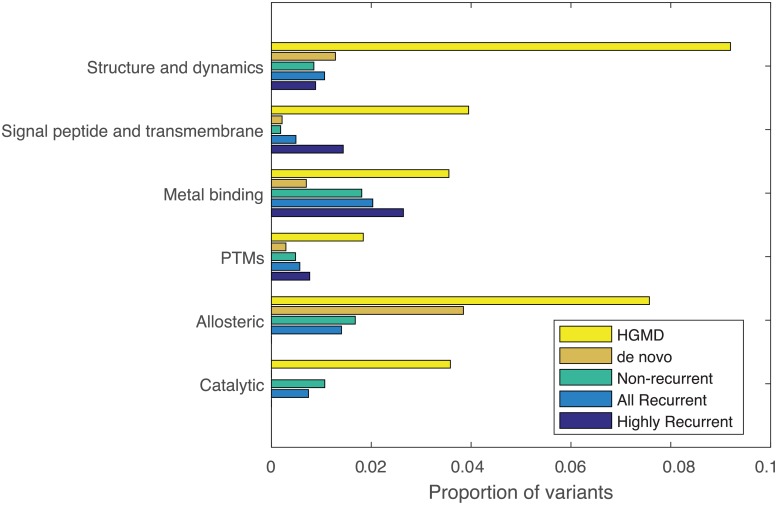
Proportion of variants predicted to impact structural and functional mechanisms among variants from single residue non-frameshifting insertion/deletion variants. A variant was considered “predicted” if its score was as high or higher than the 95-th percentile of the gnomAD score distribution. We contrast the functional impact of COSMIC, HGMD (*n* = 1556), de novo variants (*n* = 168). The highly recurrent set includes variants at residues impacted by at least 25 missense and insertion/deletion variants in the COSMIC database (*n* = 98), compared to recurrent variants which are impacted at least twice (*n* = 3622) and non-recurrent variants (*n* = 2417).

Under the rationale that repeatedly impacted residues may be a signature of selection towards tumor progression, we contrast the mechanisms frequently impacted in recurrent and non-recurrent COSMIC variants. Fig 3 highlights the proportions of variants that impact structural and functional mechanisms among de novo and COSMIC variants compared to pathogenic germline variants from HGMD. The HGMD variants more frequently effect all of the shown mechanisms, showing the most profound excess in structure and dynamics and macromolecular binding compared to the other sets. The excess of impact among HGMD variants is most likely a consequence of expert manual curation to identify pathogenic variants, whereas the set of de novo and somatic variant have undergone minor pre-processing and thereby include phenotypically neutral variation. By contrast, the de novo variants exhibit the greatest impact upon structure and dynamics, comparable to the somatic variation from COSMIC. Among the COSMIC variants, the non-recurrent set appears less frequently to impact PTM and signal peptide/transmembrane regions. Fig 3 further highlights the mechanisms exhibiting increase in functional impact among highly recurrent somatic variants including signal peptide/transmembrane regions, metal binding, and PTMs. Collectively, these results indicate the potential for predicted molecular features to identify meaningful differences in types of structural and functional features impacted by diverse sources of genetic variation.

**Fig 4:**
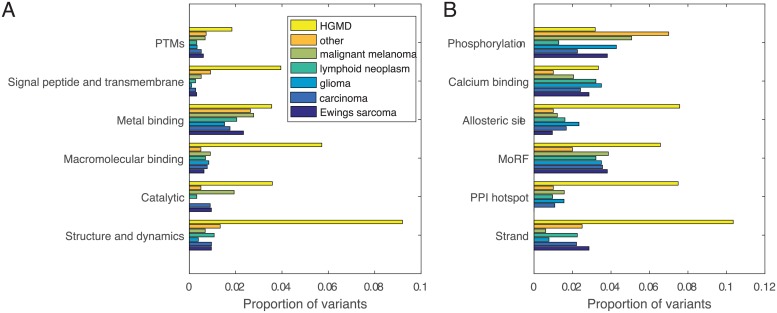
Proportion of COSMIC variants per histology type that impact structural and functional mechanisms compared to HGMD variants. (A) Changes aggregated over each class of structural and functional mechanisms and (B) Proportions for a selection of individual mechanisms.

Next, we utilize cancer histology type to identify more detailed representation of the structural and functional impact of COSMIC variation. The proportion of function-impacting somatic variants identified per histology type are shown in Fig 4A. The variability may reflect mechanisms associated with particular cancer types, such as the disparity in impact upon catalytic sites in malignant melanoma. The proportion of variants impacted by specific structural and functional mechanisms are shown in Fig 4B. Consistent with previous work [30–32], we find an excess of somatic variants that influence phosphorylation sites compared to HGMD. Conversely, a greater proportion of germline variants impact allosteric site, PPI hotspot, MoRF, and strand secondary structure. The consistent differential impact upon functional mechanisms between germline and somatic variation suggest the utility of predicted features for a variety of applications.

### Evaluation

#### Effects of manipulating the training procedures

**Fig 5:**
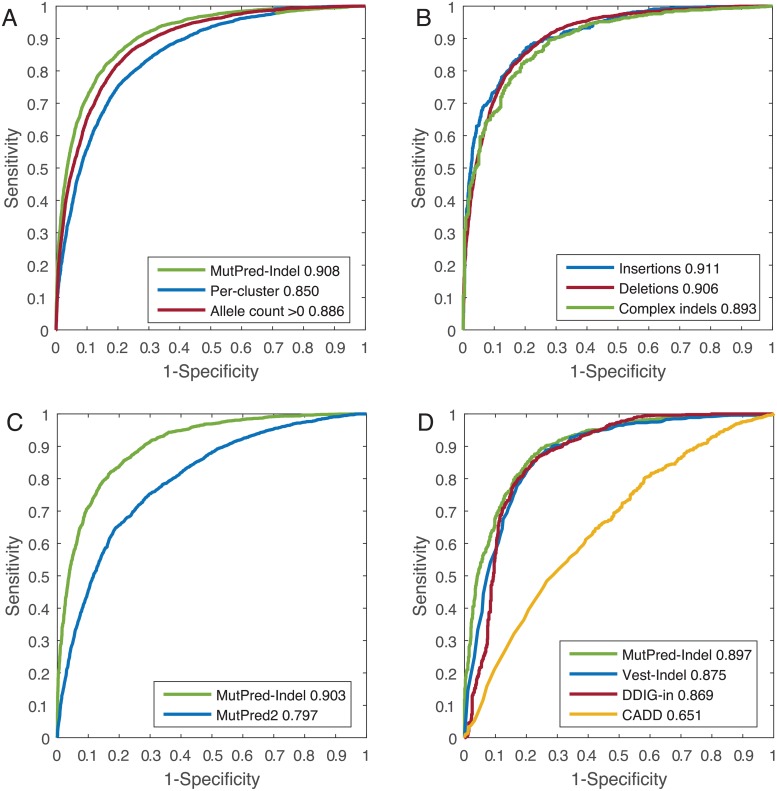
Receiver Operating Characteristic (ROC) curves and Areas Under the ROC Curves (AUC). (A) Cross-validation performance of MutPred-Indel with per-protein and per-cluster training, as well as the performance of a model with training data that includes singleton variants in gnomAD. (B) Cross-validation performance of MutPred-Indel on insertions, deletions, and complex indel variants separately. (C) Performance of MutPred-Indel and MutPred2 on single amino acid insertion/deletion variants. (D) Comparison of MutPred-Indel and three existing methods.

MutPred-Indel shows strong performance in cross-validation with the area under the ROC curve (AUC) of 0.908. Fig 5A illustrates the performance of an alternative version of the model wherein the rarest neutral variants are retained for the training set, leading to inclusion of an additional 9876 putatively neutral variants. Inclusion of these points can be interpreted as a decrease in the quality of the neutral training set by the potential inclusion of rare pathogenic variants and sequencing errors. The resultant performance exhibits only moderate decrease (AUC = 0.886). The minimal exclusion of rare gnomAD variants in the training set is intended to mitigate biases caused by the unrepresentative population structure within gnomAD and potential undersampling of various ethnic groups. Therefore, we used theoretical justification that random class label noise (e.g., sequencing errors and pathogenic variation) does not affect the optimality of the classification model [71], and included rare variation into the training set of the final model.

In Fig 5A, we illustrate the differences in performance that arise due to selection of alternate cross-validation procedure. The model trained with per-cluster cross-validation exhibits poorer performance than MutPred-Indel, which utilizes per-protein cross-validation (AUC = 0.850). The significantly reduced performance reflects the importance of gene-based features, specifically for variants in alternative protein isoforms and close homologs in the training set. The observed influence of gene-based features appear to be driven largely by alternative protein isoforms, as exhibited by the predicted performance of the model with only canonical sequence variants included in the training set, which features reduced but stable performance (AUC = 0.861).

Fig 5B shows the performance of MutPred-Indel on the subsets of insertions, deletions, and complex indel variants separately. We observe that the set of complex indel variants exhibits lower performance (AUC = 0.893) than insertions or deletions, which exhibit consistent performance (AUC = 0.911 and 0.906, respectively). If trained as three separate methods, the performance increases for complex indel variants (0.895) and decreases for insertions (0.895) and deletions (0.904). Given the similar performance and prohibitively small sample size for some types of insertion/deletion variants, we selected the collective set of variants to be the training set for MutPred-Indel. In Table 4, we further illustrate the robustness of the method utilizing the estimated performance for a model trained without each major feature set. With the exception of the gene-based FANN-GO predictions, the removal of each feature set does not significantly disrupt predictive performance of the method without feature sets described. The minimally reduced performance values listed in Table 4 justify the inclusion of these features within MutPred-Indel, without indicating any particular dominating feature that may bias performance.

**Table 4:** Performance of MutPred-Indel without key feature sets.

Feature	AUC
Conservation indexes	0.903
FANN-GO	0.871
Amino acid composition	0.905
Functional impact (*w* = 4)	0.904
Functional impact (*w* = 20)	0.904
Functional impact (entire protein)	0.905
Homolog counts	0.904

Next, we sought to ascertain whether there is an excess of high-scoring variants in genes with previously established role in cancer compared to the background of genes without strong association to cancer. We used Fisher’s exact test to compare the number of variants occurring within Tier 1 genes in the Cancer Gene Census relative to three pre-defined score thresholds for 1%, 5% and 10% FPR. For the 10% FPR threshold, we observe that 40.9% (478/1169) of high-scoring variants occur in known cancer genes compared to 27.4% (3135/11454) for genes without strong association to cancer (*P* = 3.89 ⋅ 10^−21^). For the 5% FPR threshold, we observe nearly two-fold enrichment of high-scoring variants in known cancer-associated genes compared to unassociated genes (29.9% (350/1169) compared to 16.3% (1863/11454), *P* = 5.76 ⋅ 10^−28^). The pattern is not retained for the 1% FPR score thresholds, suggesting that the 5% FPR threshold may be the optimum threshold selection for further analyses of somatic variants utilizing MutPred-Indel. These results may be indicative of the utility of MutPred-Indel for the identification of variant prioritization in somatic variation, in addition to previously established insights into impacted structural and functional mechanisms.

#### Comparison to currently existing methods

The final MutPred-Indel model represents an addition to the MutPred family of tools, including the recently updated missense predictor, MutPred2 [70]. To compare the utility of MutPred-Indel against the baseline of MutPred2, we assess the performance of the two tools on single amino acid insertion/deletion variants from the HGMD and gnomAD training sets. MutPred2 predictions for deletions were calculated as the maximum pathogenicity score over all possible missense variants at the residue impacted by the deletion. That is, the deleted residue was replaced by all other residues. These 19 variants were subsequently scored and the deletion score was reported as the maximum over these resulting scores. Similarly, MutPred2 predictions for insertions were calculated by taking the maximum missense score over all possible variants at the site of the insertion.

For the subset of single amino acid insertion/deletion variants, MutPred2 yields an AUC of 0.797 compared to 0.903 for MutPred-Indel in cross-validation (Fig 5). Despite the moderate performance of MutPred2, the superior performance and faster run time of MutPred-Indel supports the development of a distinct method designed specifically to evaluate insertion/deletion variants.

To enable direct comparison between MutPred-Indel and other methods designed to assess the pathogenicity of insertion/deletion variants, we assessed performance of each method on a random subset of 1000 pathogenic and 1000 neutral variants from the original training data, shown in Fig 5. The set of variants has been filtered to remove the training set of the methods with publicly available training data (VEST-Indel and DDIG-in). For this comparison, we extract the pathogenicity predictions generated by MutPred-Indel in cross-validation, such that neither the particular variant nor any other variant within that protein have been considered. Despite this disadvantage, we find that MutPred-Indel has the highest performance on this test set (AUC = 0.897), followed by VEST-Indel (AUC = 0.875) and DDIG-in (AUC = 0.869). For the task of discriminating between pathogenic and putatively neutral variation, MutPred-Indel shows superior performance compared to currently available methods designed to assess insertion/deletion variation.

#### Distribution of pathogenicity scores

**Fig 6:**
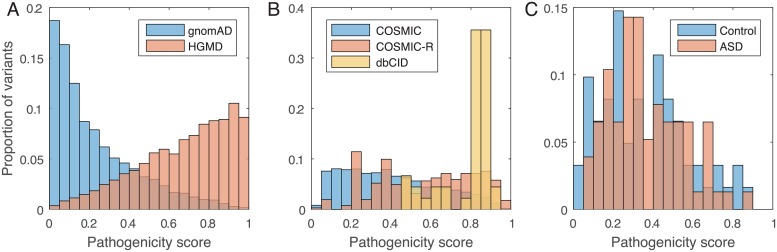
Histogram of predicted pathogenicity scores for (A) the training data using cross-validation, (B) cancer driver mutations from dbCID (yellow), highly recurrent variants (COSMIC-R, red) compared to the background in COSMIC (blue), (C) de novo non-frameshifting insertion/deletion variants in individuals with autism spectrum disorder (ASD, red) and de novo variation from unaffected siblings (Control, blue).

Fig 6A shows the distribution of prediction scores determined in cross-validation for the training data. The substantial overlap between distributions shows potential misclassifications in the training data, and justifies the use of alternate pathogenicity thresholds. To ascertain the utility of pathogenicity scores in interpreting somatic variation, Fig 6B shows the pathogenicity score distribution of recurrent somatic variation from COSMIC. In particular, we contrast the score distribution for the 5% most recurrent variants (COSMIC-R) against the remaining insertion/deletion variants in COSMIC. The distribution of scores for somatic variants is visually similar to the gnomAD distribution in Fig 6A, suggesting that a large proportion of the variants may be phenotypically neutral in isolation. The COSMIC-R variants tend to have higher pathogenicity scores with more uniform distribution, reflecting a higher proportion of damaging variants among recurrent somatic variation. Finally, we observe that the majority of dbCID cancer driver indels have pathogenicity score greater than 0.7, pointing towards the utility of MutPred-Indel in the prioritization of driver indels.

Fig 6C shows the pathogenicity score distributions for de novo insertion/deletion variants from individuals with ASD and their unaffected siblings (Control). In contrast to previous findings on loss-of-function variants in neurodevelopmental disorders [72], we do not observe an excess of high-scoring variants in individuals with ASD compared to controls. The discrepancy may suggest the reduced influence of insertion/deletion variants in this data set as a consequence of low sample size, and support the importance of de novo loss-of-function variants in neurodevelopmental disorders. Further, the spread of observed pathogenicity scores in this set supports potential utility of MutPred-Indel in the identification of impactful de novo variants. Despite the lack of strong signal for insertion/deletion variants in ASD overall, some high-scoring insertion/deletion variants in genes that carry additional loss-of-function mutations should be noted. For example, MutPred-Indel assigned high pathogenicity score to a likely pathogenic 3bp deletion (F1396/del) adjacent to the active site in the JmjC domain of lysine-specific histone demethylase *KDM6B*. It has been shown that mutations H1390/E1392 of *KDM6B* abolish lysine-specific histone demethylase activity [73]. Furthermore, the *KDM6B* gene carries three additional de novo loss-of-function mutations in ASD patients [74, 75], and three de novo missense mutations (one in ASD patient and two in the patients with intellectual disability) [76, 77], with no mutations observed in controls.

## Discussion

The wealth of variation in an individual genome necessitates computational methods to prioritize phenotypically impactful variants. In this work, we utilized computational predictors of structural and functional features to identify mechanisms impacted frequently among de novo, somatic, and germline non-frameshifting insertion/deletion variants. In addition, we developed a machine learning method to assess non-frameshifting insertion/deletion variants based upon evolutionary conservation, sequence-level, and predicted molecular features. The method, MutPred-Indel, predicts both variant pathogenicity and the types of structural and functional mechanisms impacted by individual sequence-retaining insertion/deletion variants. We show that the method has the ability to differentiate disease-causing from putatively neutral variation, and infer functionally impacted residues among diverse sources of genetic variation. The work serves to extend the MutPred family of tools, including previously developed variant effect predictors for missense [6, 70], splice [78], frameshifting and stop variants [72] allowing for targeted assessment of insertion/deletion variation to facilitate precision medicine.

A large proportion of previously developed methods to evaluate insertion/deletion variation are based upon training data that may not appropriately reflect the variation within an individual genome due to filtering based upon global or population-specific allele frequency. Stringent data cleaning procedures can result in methods that do not appropriately recognize private neutral variation, or variants that have been called in error. Detection of insertion/deletion variants is more error-prone than single nucleotide substitutions, partly due to ambiguity of mapping in repeat regions [79]. The number of insertion/deletion variants called from an individual genome may vary considerably between sequencing platforms, with concordance estimated to be as low as 57% [80]. As sequencing technologies move towards longer reads, the error rate may decrease [81]. Although both HGMD and gnomAD undergo conservative filtering procedures to reduce false calls, a nontrivial number of sequencing errors may persist. Removal of variants with low allele frequency may reduce the number of variants called in error, yet increase the potential to learn biased properties of insertion/deletion variants in training.

Development of MutPred-Indel may be influenced by methodological limitations. The training data is comprised of pathogenic and putatively neutral variation. A gold standard set of true phenotypically neutral insertion/deletion variants is not available, and the application of restrictive allele frequency thresholds may lead to systematic biases. Specifically, MutPred-Indel is trained to discriminate between disease-causing variants and putatively neutral variation found in population databases. The gnomAD database excludes individuals with severe pediatric disease and therefore the model is designed to identify variants that cause severe disease, rather than disease-associated variants. As the majority of training data impact a single residue, the number of multi-residue variants is insufficient to ascertain the suitability of MutPred-LOF to evaluate particularly lengthy alterations [72]. In this work, prediction follows the traditional framework wherein predictions are generated independently for each variant, without consideration of epistatic interactions which may substantially modify phenotypic effects.

The analyses presented here highlight the diverse spectrum of pathogenicity and functional impact attributable to non-frameshifting insertion/deletion variants. We contextualize the utility of pathogenicity prediction by presenting the apparent observable differences in score distribution among disease-causing, de novo, and recurrent somatic variation. MutPred-Indel shows robust predictive performance in cross-validation and has potential to identify pathogenicity and functional mechanisms impacted by diverse sources of genetic variation with potential utility for a variety of precision medicine applications [82, 83].
